# Supplementary material for: Integrated Metabolites and Transcriptomics at Different Growth Stages Reveal Polysaccharide and Flavonoid Biosynthesis in Cynomorium songaricum
Source: Int J Mol Sci. 2022 Sep 14;23(18):10675. doi: 10.3390/ijms231810675 (PMC9501575; doi:10.3390/ijms231810675)
Supplement: Supplementary file 1 [file ijms-23-10675-s001.zip › ijms-1894179-supplementary.pdf]

Supplementary Materials

Figure supplementary legends

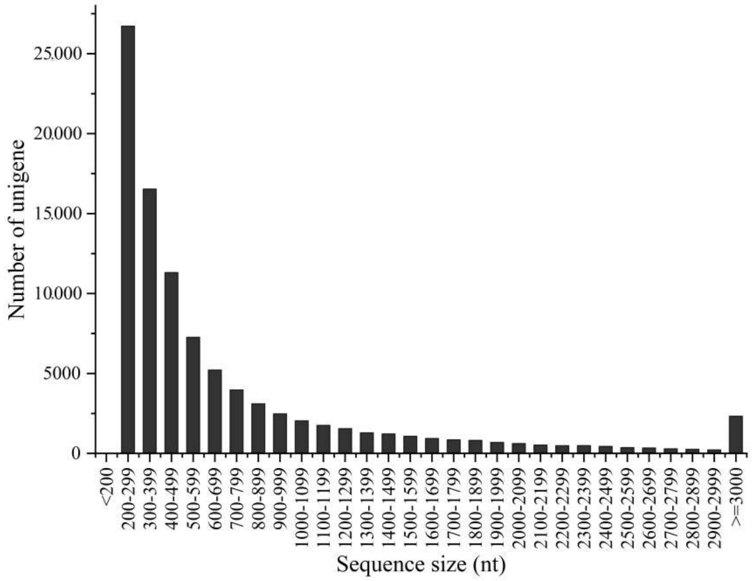

Figure S1. Length distribution of assembled unigenes.

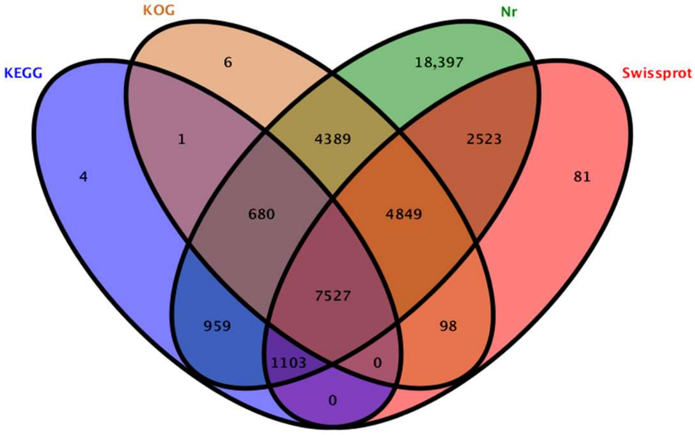

Figure S2. Basic annotation for all unigenes in *C. songaricum* on KEGG, KOG, Nr and Swissprot databases.

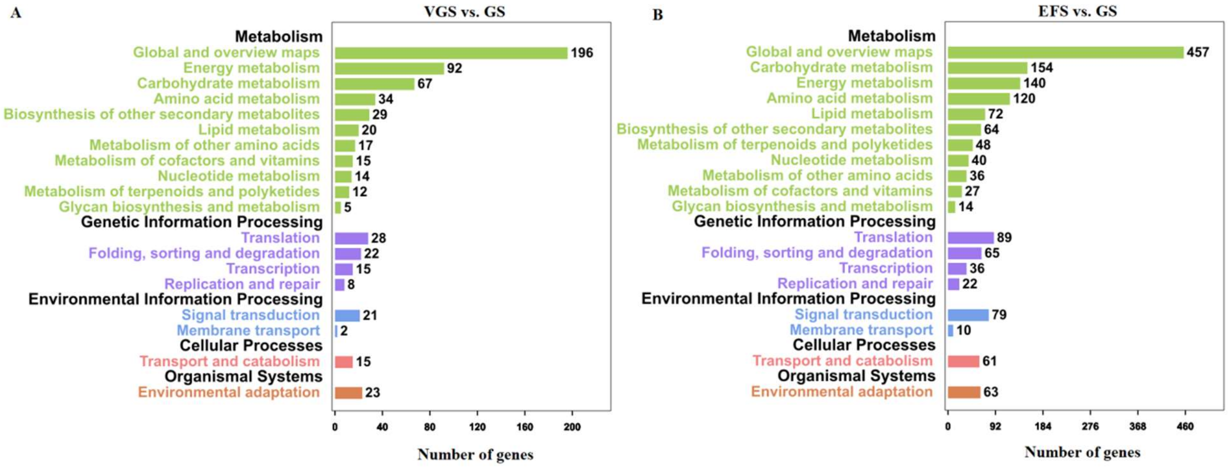

Figure S3. KEGG enrichment of DEGs at VGS vs. GS (A) and EFS vs. GS (B).

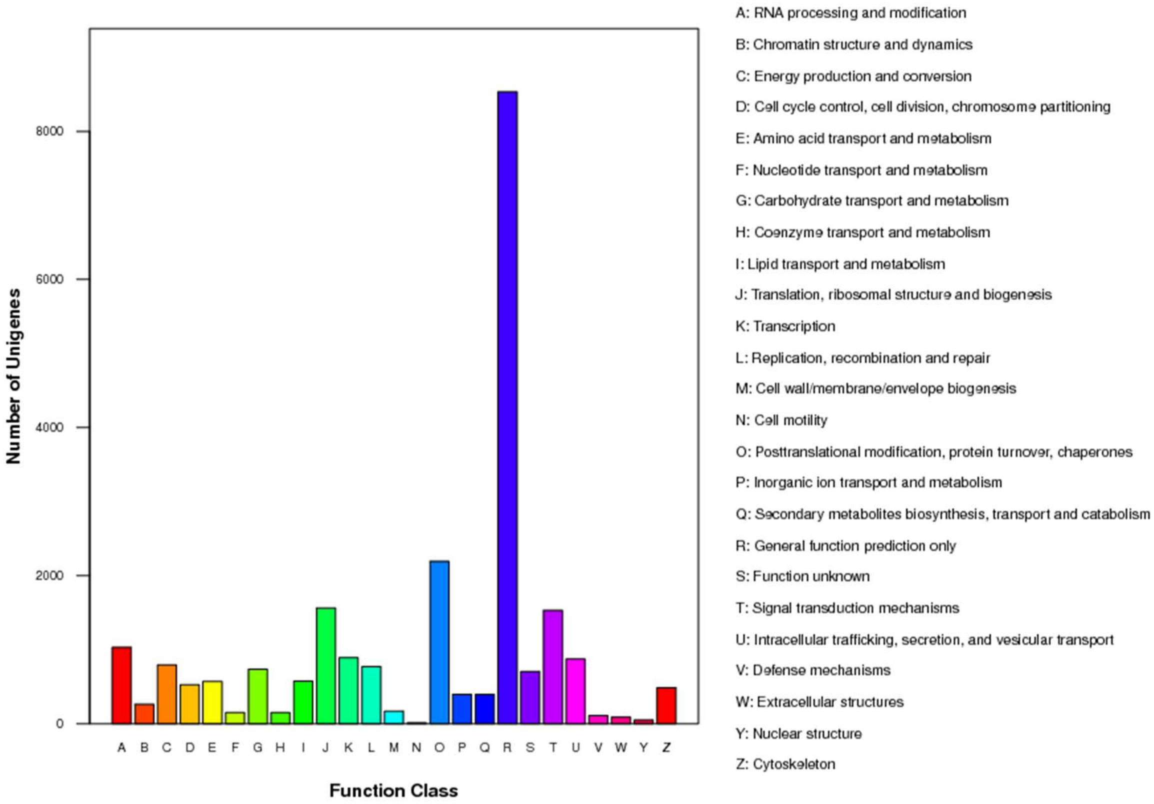

Figure S4. Distribution of unigenes in the transcriptome with KOG functional classification.

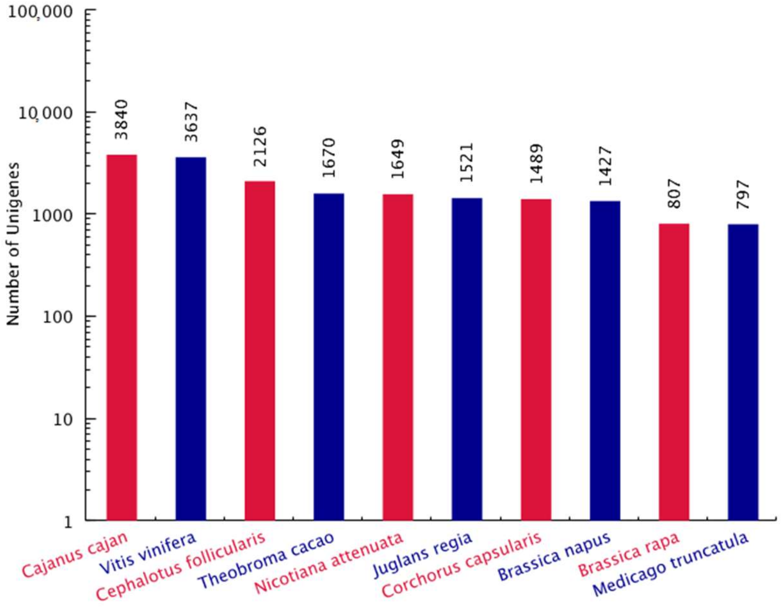

Figure S5. Annotation of unigenes on NR database and distribution of the top 10 species.

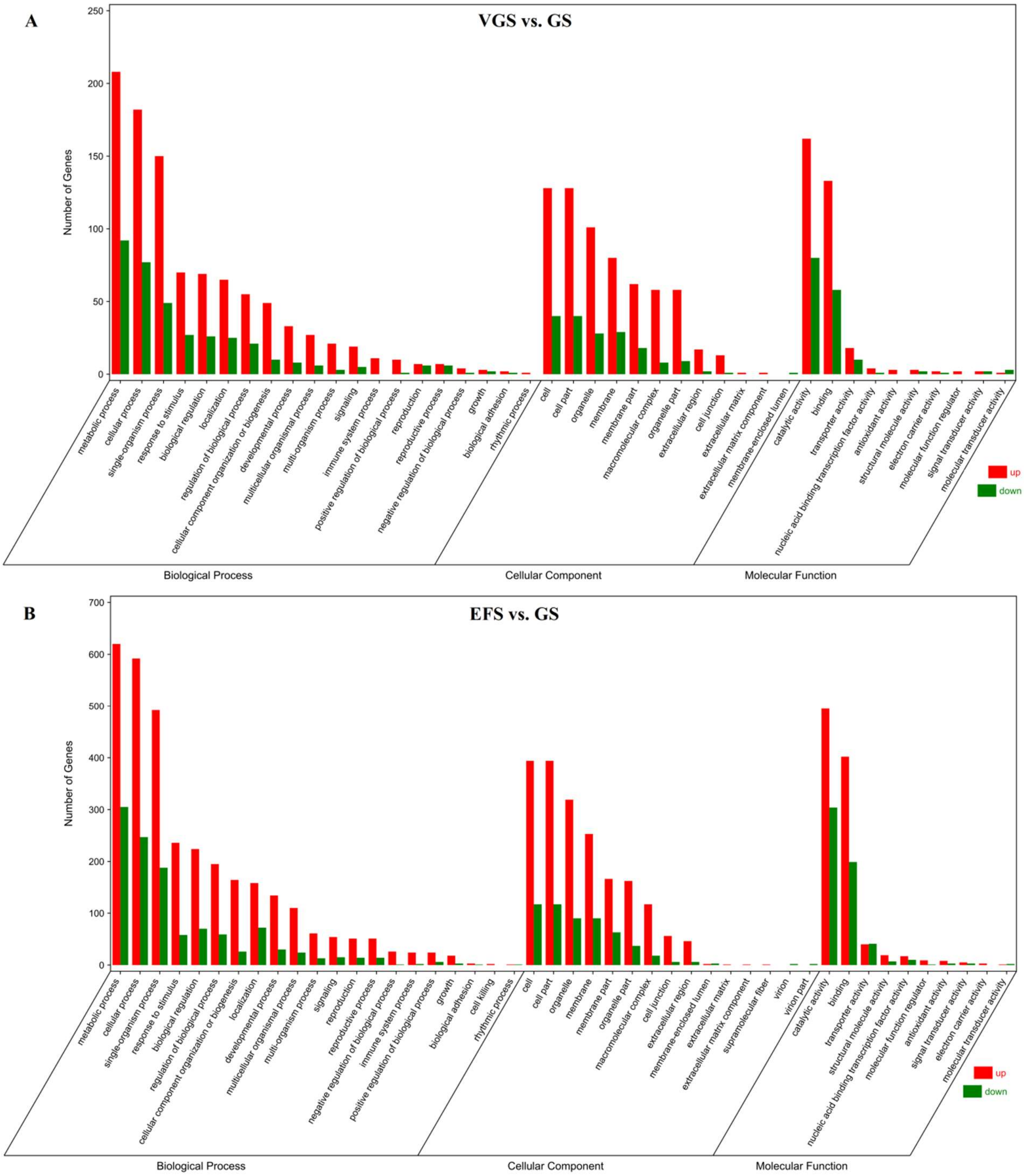

Figure S6. Annotation of unigenes on GO database and biological classification.

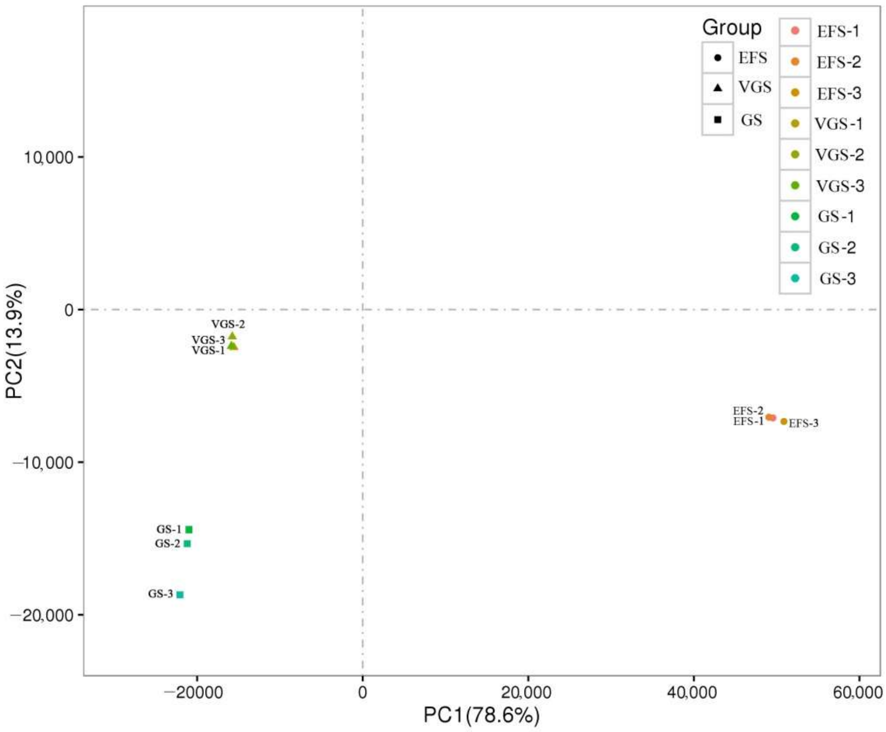

Figure S7. Principal component analysis (PCA) of GS, VGS and EFS.

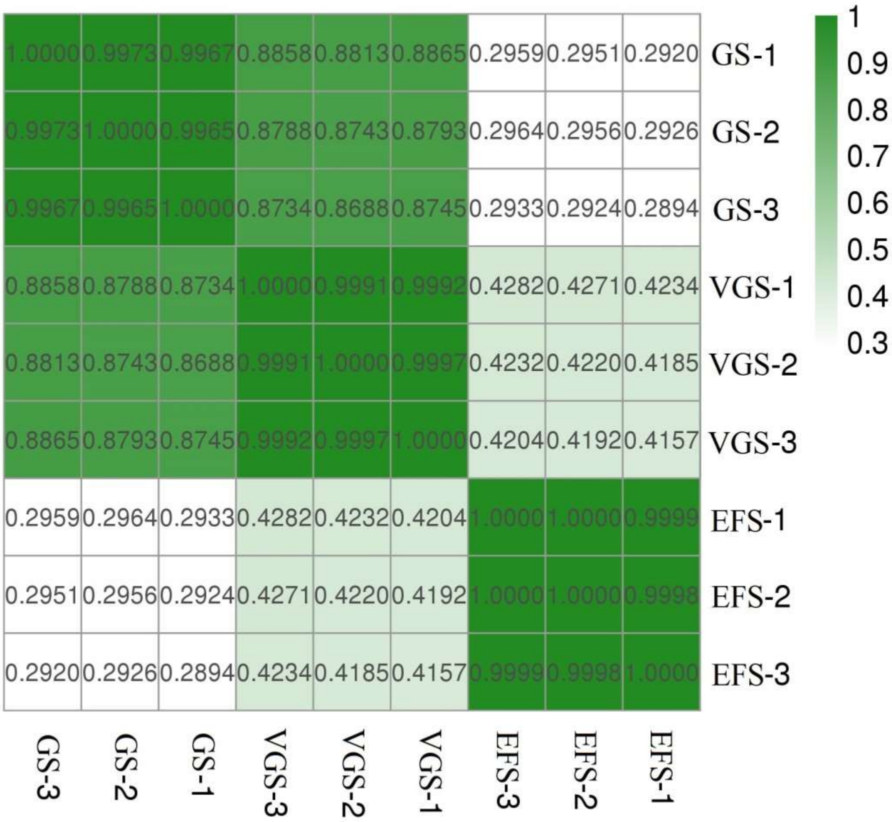

Figure S8. Pearson correlation analysis of GS, VGS and EFS.

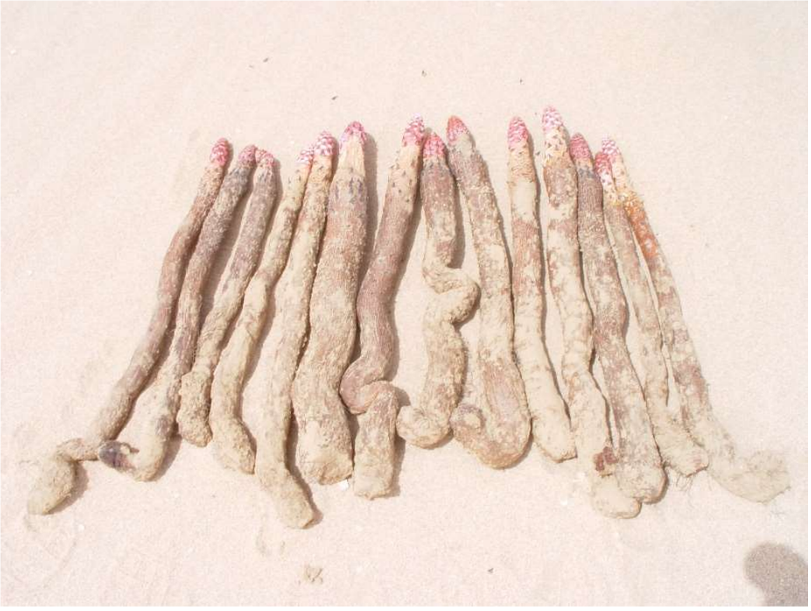

Figure S9. Growth characteristics of C. songaricum between the VGS and EFS.

Table supplementary legends

Table S1. Twenty-four genes involved in polysaccharide biosynthesis at VGS and EFS vs. GS.

Gene name	SwissProt ID	Protein name	log_2FC (VGS vs. GS)	log_2FC (EFS vs. GS)
**Glucose (5)**				
BGLU23	Q9SR37	Beta-glucosidase 23	9.74	9.87
BGLU44	Q9LV33	Beta-glucosidase 44	−1.51	−2.89
At5g56590	Q9FJU9	Glucan endo-1,3-beta-glucosidase 13	1.11	−2.73
GAPA	P09043	Glyceraldehyde-3-phosphate dehydrogenase A	9.00	9.25
MTH_209	O26311	Glyceraldehyde 3-phosphate phosphatase	1.07	1.35
**Sucrose (2)**				
INVA	P29001	Acid beta-fructofuranosidase	1.71	5.08
BFRUCT3	Q43348	Acid beta-fructofuranosidase 3	5.29	8.64
**Fructose (3)**				
FBA2	Q944G9	Fructose-bisphosphate aldolase 2, chloroplastic	6.70	5.85
PFP-BETA	P21343	Pyrophosphate--fructose 6-phosphate 1-phosphotransferase subunit beta	−1.41	−2.94
At3g55800	P46283	Sedoheptulose-1,7-bisphosphatase	7.54	7.29
**Xylan (6)**				
BXL5	Q9LJN4	Probable beta-D-xylosidase 5	1.76	1.29
GT17	Q9CA34	Probable xyloglucan galactosyltransferase GT17	−1.08	−2.43
TBL19	Q9LFT0	Protein trichome birefringence-like 19	1.14	2.45
TBL31	Q1PFD9	Protein trichome birefringence-like 31	1.78	2.50
ESK1	Q9LY46	Xylan O-acetyltransferase 1	1.25	2.34
XTH9	Q8LDW9	Xyloglucan endotransglucosylase/hydrolase protein 9	1.52	4.77
**Trehalose (1)**				
TPPF	Q9SU39	Probable trehalose-phosphate phosphatase F	1.46	4.05
**Pectin (7)**				
PAE8	Q6DBP4	Pectin acetylesterase 8	1.05	3.55
GAUT12	Q9FH36	Probable galacturonosyltransferase 12	1.06	2.16
At5g63180	Q93Z25	Probable pectate lyase 22	3.02	1.32
PME7	Q9SRX4	Probable pectinesterase/pectinesterase inhibitor 7	1.31	1.73
PMEI10	Q9SI74	Pectinesterase inhibitor 10	−1.43	1.80
PME15	Q9ZQA3	Probable pectinesterase 15	1.32	−4.61
PME40	O81301	Probable pectinesterase/pectinesterase inhibitor 40	2.47	3.17

Table S2. Fifty-five genes involved in other primary metabolism at VGS and EFS vs. GS.

Gene name	SwissProt ID	Protein name	log_2FC (VGS vs. GS)	log_2FC (EFS vs. GS)
**Lipid and fatty acid catabolic process**				
BEAT	O64988	Acetyl-CoA-benzylalcohol acetyltransferase	1.84	− 5.09
N/A	O04353	Acyl-lipid (9-3)-desaturase	1.03	1.82
AOC	A2XID3	Allene oxide cyclase, chloroplastic	1.12	− 2.86
At2g04020	Q9SIF3	GDSL esterase/lipase At2g04020	− 1.54	− 2.06
At3g26430	Q9LIN2	GDSL esterase/lipase At3g26430	8.93	4.59
At3g48460	Q9STM6	GDSL esterase/lipase At3g48460	1.12	2.09
At5g33370	Q8LB81	GDSL esterase/lipase At5g33370	2.40	7.12
GPAT5	Q9CAY3	Glycerol-3-phosphate acyltransferase 5	2.86	2.39
GDPD1	Q9SGA2	Glycerophosphodiester phosphodiesterase GDPD1, chloroplastic	3.29	3.27
NPC4	Q9SRQ7	Non-specific phospholipase C4	2.51	2.31
HB2	Q93Y92	Non-symbiotic hemoglobin 2	− 1.34	− 1.19
MATP6-A	P29527	Oleosin 18.2 kDa	2.21	1.87
At4g25140	P29525	Oleosin 18.5 kDa	5.16	5.31
MTM1	F4J3T8	Phosphatidylinositol-3-phosphatase myotubularin-1	1.66	1.73
At4g16820	O23522	Phospholipase A1-Ibeta2, chloroplastic	2.27	− 2.55

NMT2	Q944H0	Phosphomethylethanolamine N-methyltransferase	1.14	2.36
At5g60760	Q9FJH9	P-loop NTPase domain-containing protein LPA1 homolog 1	11.36	9.91
LPPB	Q9SUW4	Probable lipid phosphate phosphatase beta	− 1.06	− 1.48
CER2	Q39048	Protein ECERIFERUM 2	− 1.45	− 1.09
At1g17710	Q9FZ62	Inorganic pyrophosphatase 2	− 1.38	− 4.58
**Amino acid metabolism**				
serA	O29445	D-3-phosphoglycerate dehydrogenase	− 6.41	− 2.82
GEK1	Q9ZPQ3	D-aminoacyl-tRNA deacylase	9.41	7.71
GGAT2	Q9S7E9	Glutamate--glyoxylate aminotransferase 2	6.41	5.76
GLN1-2	Q8LCE1	Glutamine synthetase cytosolic isozyme 1-2	5.92	6.86
GLN2	O22506	Glutamine synthetase, chloroplastic	9.22	9.34
GLN4	P15102	Glutamine synthetase leaf isozyme, chloroplastic	9.84	9.84
OVA9	Q8W4F3	Glutamine--tRNA ligase, cytoplasmic	− 1.23	− 2.69
GRXC3	Q6K609	Glutaredoxin-C3	2.75	4.50
GRXC6	Q8L9S3	Glutaredoxin-C6	− 1.04	1.10
GGT3	Q9M0G0	Glutathione hydrolase 3	− 1.18	− 5.02
GSTF10	P42761	Glutathione S-transferase F10	9.15	8.81
GSTF12	Q9FE46	Glutathione S-transferase F12	3.08	2.62
DHAR2	Q67UK9	Probable glutathione S-transferase DHAR2, chloroplastic	10.97	10.98
At2g30660	Q6NMB0	Probable 3-hydroxyisobutyryl-CoA hydrolase 3	1.30	2.55
AGT1	Q56YA5	Serine--glyoxylate aminotransferase	10.27	9.65
GLYM1	P49357	Serine hydroxymethyltransferase 1, mitochondrial	9.87	9.93
GLYM2	P49358	Serine hydroxymethyltransferase 2, mitochondrial	9.87	9.62
**Others associated with carbohydrate metabolism**				
ADH2	P04707	Alcohol dehydrogenase 2	− 1.03	− 1.78
ACA7	Q8L817	Alpha carbonic anhydrase 7	− 2.56	− 1.72
CAHC	P17067	Carbonic anhydrase, chloroplastic	11.10	10.83
CTL2	Q9LSP9	Chitinase-like protein 2	− 1.13	− 2.20
ChiC	O81862	Class V chitinase	− 1.47	− 2.53
CYN	A5BJL8	Cyanate hydratase	− 1.34	− 5.26
CEL5	Q2V4L8	Endoglucanase 3	1.62	1.43
GH5FP	C0HLA0	Glycosyl hydrolase 5 family protein	1.83	5.77
HPPR	Q65CJ7	Hydroxyphenylpyruvate reductase	8.21	9.74
MDHG	P46488	Malate dehydrogenase, glyoxysomal	9.08	9.08
KPPR	P27774	Phosphoribulokinase, chloroplastic	9.60	9.17
At1g56190	P50318	Phosphoglycerate kinase 2, chloroplastic	10.39	10.28
At5g64460	Q9FGF0	Phosphoglycerate mutase-like protein 1	1.09	1.26
AKR1	C6TBN2	Probable aldo-keto reductase 1	8.64	9.26
PP2A10	Q9SY57	Protein PHLOEM PROTEIN 2-LIKE A10	− 12.07	− 9.24
SDR2b	Q9ZUH5	Short-chain dehydrogenase/reductase 2b	1.06	1.13
TKL-2	F4IW47	Transketolase-2, chloroplastic	6.08	5.48
TKT3	Q42676	Transketolase, chloroplastic	8.79	9.38

Table S3. Eleven genes involved in flavonoid biosynthesis at VGS and EFS vs. GS.

Gene name	SwissProt ID	Protein name	log_2FC (VGS vs. GS)	log_2FC (EFS vs. GS)
4CLL1	Q9LQ12	4-coumarate-CoA ligase-like 1	1.45	1.73
4CLL6	Q84P24	4-coumarate-CoA ligase-like 6	3.33	8.48
HST	Q9FI78	Shikimate O-hydroxycinnamoyltransferase	1.67	2.09
CHI3	Q8VZW3	Probable chalcone--flavanone isomerase 3	1.57	1.24
CAD9	P42734	Probable cinnamyl alcohol dehydrogenase 9	1.10	− 1.70
CYP714C2	Q2QYH7	Cytochrome P450 714C2	1.45	1.59
CYP93B1	P93149	Licodione synthase	1.22	1.42
F6'H1	Q9LHN8	Feruloyl CoA ortho-hydroxylase 1	2.11	− 11.78
UGT84A13	V5LLZ9	Gallate 1-beta-glucosyltransferase	2.52	2.02
UGT87A1	O64732	UDP-glycosyltransferase 87A1	− 1.97	-3.00
UGT94E5	F8WKW8	Beta-D-glucosyl crocetin beta-1,6-glucosyltransferase	1.09	− 4.50

Table S4. Five genes involved in terpene biosynthesis at VGS and EFS vs. GS.

Gene name	SwissProt ID	Protein name	log_2FC (VGS vs. GS)	log_2FC (EFS vs. GS)

PINS	O23945	(-)-alpha-pinene synthase	7.16	5.41
CYP716A15	F6H9N6	Beta-amyrin 28-monooxygenase	1.08	− 3.20
CYP716A52v2	I7C6E8	Beta-amyrin 28-monooxygenase	2.27	5.02
GGR	Q39108	Heterodimeric geranylgeranyl pyrophosphate synthase small subunit	1.38	2.55
STPS1	B5A435	Sesquiterpene synthase	8.57	6.09

Table S5. Twenty genes involved in cell growth and flower development at VGS and EFS vs. GS.

**Gene name**	**SwissProt ID**	**Protein name**	**log_2FC (VGS vs. GS)**	**log_2FC (EFS vs. GS)**
**Cell growth (9)**				
AC58	P30167	Actin-58	8.64	8.99
ADF	P30175	Actin-depolymerizing factor	1.06	1.65
ATJ11	Q9FYB5	Chaperone protein dnaJ 11	− 1.10	1.88
CYCP3-1	Q75HV0	Cyclin-P3-1	1.19	2.77
SDS	Q1PFW3	Cyclin-SDS	4.69	5.72
LRX6	Q9LUI1	Leucine-rich repeat extensin-like protein 6	1.51	1.85
MIZ1	O22227	Protein MIZU-KUSSEI 1	− 1.43	− 1.06
PATROL1	Q8RX56	Protein unc-13 homolog	− 1.16	− 1.35
TBB7	Q6VAF5	Tubulin beta-7 chain	9.49	9.43
**Flower development (11)**				
CSLD4	Q9SZL9	Cellulose synthase-like protein D4	− 3.38	− 1.88
EXLB1	O23547	Expansin-like B1	− 1.05	− 2.71
AP1	P35631	Floral homeotic protein APETALA 1	2.74	8.25
ASOL	P29162	L-ascorbate oxidase homolog	1.19	2.88
AMP1	Q9M1S8	Probable glutamate carboxypeptidase AMP1	− 1.02	− 4.74
TKPR2	Q9CA28	Tetraketide alpha-pyrone reductase 2	1.23	1.34
TCTP1	P31265	Translationally-controlled tumor protein 1	9.23	10.45
CYP704B1	Q9C788	Cytochrome P450 704B1	1.86	1.92
HAT	Q9M2N5	Zinc finger BED domain-containing protein DAYSLEEPER	− 7.25	− 4.08
Os05g0239150	B9FJG3	Zinc finger BED domain-containing protein RICESLEEPER 1	− 1.62	− 1.44
Os05g0583200	Q75HY5	Zinc finger BED domain-containing protein RICESLEEPER 3	2.13	2.50

Table S6. Eight genes involved in other cell morphogenesis at VGS and EFS vs. GS.

**Gene name**	**SwissProt ID**	**Protein name**	**log_2FC (VGS vs. GS)**	**log_2FC (EFS vs. GS)**
N/A	Q9XHP0	11S globulin seed storage protein 2	− 7.63	− 3.80
AT2S1	P15457	2S seed storage protein 1	10.08	10.97
CRA1	P15455	12S seed storage protein CRA1	3.64	4.26
CRB	P15456	12S seed storage protein CRB	4.49	5.13
SOP1	Q9SQ57	Peroxygenase	4.98	4.19
SBT1.3	Q9FLI4	Subtilisin-like protease SBT1.3	− 1.72	− 4.13
SBT1.7	O65351	Subtilisin-like protease SBT1.7	− 2.90	− 3.08
CYSEP	O65039	Vignain	2.27	4.59

Table S7. Twenty-two genes involved in hormone biosynthesis and signaling at VGS and EFS vs. GS.

**Gene name**	**SwissProt ID**	**Protein name**	**log_2FC (VGS vs. GS)**	**log_2FC (EFS vs. GS)**
**Hormone biosynthesis (7)**				
YUC8	Q9SVU0	Probable indole-3-pyruvate monooxygenase YUCCA8	1.31	1.72
AIPT	Q5GHF7	Adenylate isopentenyltransferase	1.94	2.73
LOG5	Q8LBB7	Cytokinin riboside 5'-monophosphate phosphoribohydrolase LOG5	1.30	1.14
LE	O24648	Gibberellin 3-beta-dioxygenase 1	− 2.11	− 3.91
ACO1	Q9MB94	1-aminocyclopropane-1-carboxylate oxidase	1.01	− 3.54
ACS1	P31531	1-aminocyclopropane-1-carboxylate synthase	1.07	− 2.70
CYP707A6	Q09J78	Abscisic acid 8'-hydroxylase 2	− 1.07	− 2.02

**Hormone signaling (15)**				
AUX22D	O24542	Auxin-induced protein 22D	1.18	3.68
ARP12.5	Q05349	Auxin-repressed 12.5 kDa protein	− 1.83	− 2.75
SAUR71	Q9SGU2	Auxin-responsive protein SAUR71	3.44	3.82
GH3.6	Q9LSQ4	Indole-3-acetic acid-amido synthetase GH3.6	2.43	2.58
PILS2	Q9C999	Protein PIN-LIKES 2	− 2.00	− 4.95
AHK4	Q9C5U0	Histidine kinase 4	4.25	3.47
JOX2	Q9FFF6	Jasmonate-induced oxygenase 2	1.52	− 7.98
AIL1	Q1PFE1	AP2-like ethylene-responsive transcription factor AIL1	− 1.63	1.23
AIL6	Q52QU2	AP2-like ethylene-responsive transcription factor AIL6	− 1.74	1.43
ETR2	Q0WPQ2	Ethylene receptor 2	− 1.03	− 1.95
SHN3	Q3E958	Ethylene-responsive transcription factor SHINE 3	− 1.10	− 1.97
REF6	Q9STM3	Lysine-specific demethylase REF6	4.79	3.80
ERF010	Q9FH94	Ethylene-responsive transcription factor ERF010	− 1.69	− 4.43
ERF034	Q8LBQ7	Ethylene-responsive transcription factor ERF034	− 1.12	1.49
ERF114	Q9FH54	Ethylene-responsive transcription factor ERF114	3.01	4.33

Table S8. Nineteen genes involved in other bio-signaling at VGS and EFS vs. GS.

Gene name	SwissProt ID	Protein name	log_2FC (VGS vs. GS)	log_2FC (EFS vs. GS)
**Protein kinase**				
KRP7	Q94CL9	Cyclin-dependent kinase inhibitor 7	1.04	3.62
CRK36	Q9XEC6	Cysteine-rich receptor-like protein kinase 36	2.31	3.69
LECRK2	A2XQD3	G-type lectin S-receptor-like serine/threonine-protein kinase LECRK2	− 2.01	− 7.27
ERECTA	Q42371	LRR receptor-like serine/threonine-protein kinase ERECTA	− 1.07	1.95
SERPINE1	P13909	Plasminogen activator inhibitor 1	− 9.18	− 9.18
At1g35710	Q9LP24	Probable leucine-rich repeat receptor-like protein kinase At1g35710	− 5.21	− 5.59
At5g18500	Q8LEB6	Probable receptor-like protein kinase At5g18500	1.26	2.95
PERK4	Q9ZNQ8	Proline-rich receptor-like protein kinase PERK4	− 1.11	− 3.82
TMK4	Q9LK43	Receptor-like kinase TMK4	1.98	1.07
SCPL31	O04084	Serine carboxypeptidase-like 31	− 2.83	− 3.04
SCPL40	Q0WRX3	Serine carboxypeptidase-like 40	− 4.44	− 6.83
UNC	Q9SYB9	Serine/threonine-protein kinase UCN	− 2.14	− 2.48
**Calcium**				
RALFL4	Q9FZA0	Protein RALF-like 4	1.59	1.20
**Others**				
GF14C	Q6ZKC0	14-3-3-like protein GF14-C	6.78	5.98
CLE3	Q3EDH8	CLAVATA3/ESR (CLE)-related protein 3	− 1.89	− 1.28
CLE27	Q9LUA1	CLAVATA3/ESR (CLE)-related protein 27	− 1.53	− 1.37
TIMP1	P20414	Metalloproteinase inhibitor 1	− 10.05	− 10.05
ADM	O62827	Pro-adrenomedullin	− 9.72	− 5.21
At4g32285	Q8S9J8	Probable clathrin assembly protein At4g32285	2.16	3.28

Table S9. Twenty-eight TFs involved in flavonoid biosynthesis as well as growth and development at VGS and EFS vs. GS.

Gene name	SwissProt ID	Protein name	log_2FC (VGS vs. GS)	log_2FC (EFS vs. GS)
**Flavonoid biosynthesis (12)**				
**MYB (6)**				
LIMYB	Q9FFJ8	L10-interacting MYB domain-containing protein	1.45	− 3.00
MYB2	Q10MB4	Transcription factor MYB2	− 5.00	− 11.58
MYB14	Q9SJX8	Transcription factor MYB14	2.21	− 2.24
MYB52	Q6R0C4	Transcription factor MYB52	1.66	2.56
MYB83	Q9C6U1	Transcription factor MYB83	1.22	2.58
MYB306	P81392	Myb-related protein 306	1.37	1.64
**bHLH (2)**				
BHLH52	Q9SA82	Transcription factor bHLH52	2.78	4.75
BHLH94	Q9SK91	Transcription factor bHLH94	1.31	− 1.83

**WRKY (4)**				
WRKY6	Q9C519	WRKY transcription factor 6	1.66	− 1.52
WRKY53	Q9SUP6	Probable WRKY transcription factor 53	1.57	2.26
WRKY70	Q9LY00	Probable WRKY transcription factor 70	− 1.20	− 3.28
WRKY72	Q9LXG8	Probable WRKY transcription factor 72	− 3.38	− 1.24
**Growth and development (16)**				
**Cell growth (8)**				
DREB2D	Q9LQZ2	Dehydration-responsive element-binding protein 2D	1.03	− 1.86
DREB3	Q9LYD3	Dehydration-responsive element-binding protein 3	1.82	2.40
At2g01810	Q9ZUA9	PHD finger protein At2g01810	− 1.74	− 3.64
MMD1	Q7X6Y7	PHD finger protein MALE MEIOCYTE DEATH 1	− 1.26	− 2.55
PRE5	Q9LJX1	Transcription factor PRE5	2.72	6.87
TCP9	O64647	Transcription factor TCP9	2.19	2.94
TCP18	A1YKT1	Transcription factor TCP18	− 1.07	− 1.29
UPB1	O22901	Transcription factor UPBEAT1	1.68	2.19
**Flower development (8)**				
AHL17	Q9LTA2	AT-hook motif nuclear-localized protein 17	− 1.00	− 4.88
AHL20	Q8GWQ2	AT-hook motif nuclear-localized protein 20	− 2.43	− 9.22
AHL22	O22130	AT-hook motif nuclear-localized protein 22	− 1.28	− 2.70
AHL23	O23620	AT-hook motif nuclear-localized protein 23	− 1.23	− 3.81
MIP1B	Q9LRM4	B-box domain protein 31	2.02	1.75
HEC2	Q9SND4	Transcription factor HEC2	− 2.58	− 4.44
SCRM	Q9LSE2	Transcription factor ICE1	1.46	3.35
PAN	Q9SX27	Transcription factor PERIANTHIA	− 2.45	− 2.20

Table S10. Ten genes involved in other TFs at VGS and EFS vs. GS.

Gene name	SwissProt ID	Protein name	log_2FC (VGS vs. GS)	log_2FC (EFS vs. GS)
**B3 domain-containing protein**				
ARF31	Q8GYJ2	B3 domain-containing protein At2g36080	− 2.26	− 1.67
Os03g0120900	Q8LMR9	B3 domain-containing protein Os03g0120900	1.85	2.19
Os12g0591300	Q2QMT7	B3 domain-containing protein Os12g0591400	1.19	− 1.91
**Transcription repressor**				
OFP7	Q9ZU65	Transcription repressor OFP7	2.80	1.08
OFP11	O23341	Transcription repressor OFP11	1.18	1.35
OFP14	Q9S7T5	Transcription repressor OFP14	− 1.41	− 2.75
**Zipper protein**				
ATHB-40	O23208	Homeobox-leucine zipper protein ATHB-40	− 1.82	− 1.99
At1g68200	Q9C9F5	Zinc finger CCCH domain-containing protein 15	1.76	4.08
At2g19810	O82199	Zinc finger CCCH domain-containing protein 20	− 1.49	− 2.10
ZHD1	A2Z259	Zinc-finger homeodomain protein 1	− 2.82	2.22

Table S11. Six genes involved in polysaccharide transport at VGS and EFS vs. GS.

Gene name	SwissProt ID	Protein name	log_2FC (VGS vs. GS)	log_2FC (EFS vs. GS)
**Sugar transporter (3)**				
SWEET5	Q9FM10	Bidirectional sugar transporter SWEET5	6.27	7.12
slc37a2	Q7SY29	Glucose-6-phosphate exchanger SLC37A2	10.56	8.73
At5g55950	Q9FG70	Nucleotide-sugar uncharacterized transporter 2	1.03	1.13
**ABC transporter family (3)**				
ABCB2	Q8LPK2	ABC transporter B family member 2	6.70	3.86
ABCG1	O80946	ABC transporter G family member 1	− 1.08	− 1.08
ABCG22	Q93YS4	ABC transporter G family member 22	− 1.28	− 1.17

Table S12. Thirty-three genes involved in other transport at VGS and EFS vs. GS.

Gene name	SwissProt ID	Protein name	log_2FC (VGS vs. GS)	log_2FC (EFS vs. GS)
**Protein transport**				

ATG16L1	Q5RAC9	Autophagy-related protein 16-1	1.04	1.67
PEX12	Q9M841	Peroxisome biogenesis protein 12	− 10.61	− 5.76
SFH12	Q94A34	Phosphatidylinositol/phosphatidylcholine transfer protein SFH12	2.04	1.69
**Lipid transport**				
LTP	Q39794	Non-specific lipid-transfer protein	− 3.61	− 1.36
LTP4	Q9LLR6	Non-specific lipid-transfer protein 4	9.47	9.93
**Amino acid transport**				
CAT1	Q84MA5	Cationic amino acid transporter 1	2.16	3.49
BAC2	Q9CA93	Mitochondrial arginine transporter BAC2	− 2.28	− 3.89
**Aquaporin**				
PIP2-2	P43287	Aquaporin PIP2-2	3.01	3.71
TIP1-2	Q41963	Aquaporin TIP1-2	8.55	9.36
TIP2-1	Q41951	Aquaporin TIP2-1	10.40	10.39
TIP1-1	P50156	Probable aquaporin TIP1-1	− 9.87	− 9.87
**Ion transport**				
CHX17	Q9SUQ7	Cation/H(+) antiporter 17	− 3.63	− 3.81
IRT2	Q6L8G1	Fe(2+) transport protein 2	− 4.54	− 1.87
PHT1-4	Q96303	Inorganic phosphate transporter 1-4	1.62	2.88
MTP4	Q10PP8	Metal tolerance protein 4	3.50	3.22
MT4B	Q42377	Metallothionein-like protein 4B	− 6.14	− 3.06
MTI	P30564	Metallothionein-like protein type 2	− 5.55	− 11.82
NDT1	O22261	Nicotinamide adenine dinucleotide transporter 1	1.30	1.13
CML18	Q9M8U1	Probable calcium-binding protein CML18	− 2.77	− 4.27
NHX1	Q68KI4	Sodium/hydrogen exchanger 1	1.43	1.28
CAX1	Q39253	Vacuolar cation/proton exchanger 1	9.34	8.96
ZIP3	Q7XLD4	Zinc transporter 3	− 1.07	− 2.95
**Transmembrane transport**				
DTX40	Q9LVD9	Protein DETOXIFICATION 40	− 1.22	− 6.56
DTX45	Q9SVE7	Protein DETOXIFICATION 45	2.35	1.66
At4g08290	Q9SUF1	WAT1-related protein At4g08290	− 2.51	− 8.35
**Oligopeptide transport**				
NPF4.3	Q93VV5	Protein NRT1/ PTR FAMILY 4.3	− 1.01	− 5.37
NPF5.6	P0CI03	Protein NRT1/ PTR FAMILY 5.6	1.54	− 1.67
NPF6.2	Q9SZY4	Protein NRT1/ PTR FAMILY 6.2	− 1.01	− 1.22
**Virus transport**				
POL	P19199	Polyprotein P3	− 1.34	− 1.10
POL2	Q9YK98	RNA2 polyprotein	1.04	− 1.17
**Others**				
XI-E	F4HWY6	Myosin-11	− 1.12	− 1.33
ORP1C	Q8L751	Oxysterol-binding protein-related protein 1C	− 1.10	− 1.23
At3g12180	Q9C7D7	Protein cornichon homolog 1	1.76	1.62

Table S13. Forty-three genes involved in energy and photosynthesis at VGS and EFS vs. GS.

Gene name	SwissProt ID	Protein name	log_2FC (VGS vs. GS)	log_2FC (EFS vs. GS)
**Energy**				
ATPD	Q9SSS9	ATP synthase subunit delta, chloroplastic	10.03	9.44
ATPC	P29790	ATP synthase gamma chain, chloroplastic	9.33	8.37
petC	P26291	Cytochrome b6-f complex iron-sulfur subunit, chloroplastic	9.32	9.47
MT-CO1	Q6EMS9	Cytochrome c oxidase subunit 1	− 10.83	− 7.36
COX6B-1	Q9S7L9	Cytochrome c oxidase subunit 6b-1	11.01	10.41
SEND33	Q43517	Ferredoxin-1, chloroplastic	11.38	10.90
PETH	O04977	Ferredoxin--NADP reductase, leaf-type isozyme, chloroplastic	5.03	5.38
GAPN	P93338	NADP-dependent glyceraldehyde-3-phosphate dehydrogenase	2.27	1.16
RCA1	Q7X9A0	Ribulose bisphosphate carboxylase/oxygenase activase 1, chloroplastic	8.75	8.31
RCA2	Q7X999	Ribulose bisphosphate carboxylase/oxygenase activase 2, chloroplastic	12.79	12.35
RBCS	P31333	Ribulose bisphosphate carboxylase small subunit, chloroplastic	14.83	14.67
PETE	P00299	Plastocyanin A, chloroplastic	12.18	12.32
CYB561D	Q9C540	Probable transmembrane ascorbate ferrireductase 4	1.29	2.57
**Photosynthesis**				
LHCA4	P27521	Chlorophyll a-b binding protein 4, chloroplastic	10.05	9.80
LHCA1	Q01667	Chlorophyll a-b binding protein 6, chloroplastic	10.22	9.89

CAB7	P10708	Chlorophyll a-b binding protein 7, chloroplastic	10.43	9.82
CAB13	P27489	Chlorophyll a-b binding protein 13, chloroplastic	8.91	8.90
CAB21	P27493	Chlorophyll a-b binding protein 21, chloroplastic	6.63	6.52
CAB-151	P27518	Chlorophyll a-b binding protein 151, chloroplastic	11.99	11.71
CAP10A	P27524	Chlorophyll a-b binding protein CP24 10A, chloroplastic	10.78	10.30
LHCB4.1	Q07473	Chlorophyll a-b binding protein CP29.1, chloroplastic	7.62	7.12
HPR-A	P13443	Glycerate dehydrogenase	8.85	9.18
CRD1	Q6SJV8	Magnesium-protoporphyrin IX monomethyl ester [oxidative] cyclase, chloroplastic	9.23	8.96
PSBO	Q40459	Oxygen-evolving enhancer protein 1, chloroplastic	11.47	10.89
PSBP1	Q7DM39	Oxygen-evolving enhancer protein 2-1, chloroplastic	11.04	11.04
PSBQ	P12301	Oxygen-evolving enhancer protein 3, chloroplastic	10.66	10.44
LHCA3	Q9SY97	Photosystem I chlorophyll a/b-binding protein 3-1, chloroplastic	10.83	10.22
psaD	P12353	Photosystem I reaction center subunit II, chloroplastic	10.21	9.73
PSAF	Q9SHE8	Photosystem I reaction center subunit III, chloroplastic	10.06	9.48
PSAE	P13194	Photosystem I reaction center subunit IV, chloroplastic	10.20	9.66
PSAEB	Q41229	Photosystem I reaction center subunit IV B, chloroplastic	9.49	9.75
PSAG	Q9S7N7	Photosystem I reaction center subunit V, chloroplastic	10.61	9.96
PSAH	O04006	Photosystem I reaction center subunit VI, chloroplastic	6.73	7.06
PSAN	P49107	Photosystem I reaction center subunit N, chloroplastic	5.68	5.60
PSAK	Q9SUI5	Photosystem I reaction center subunit psaK, chloroplastic	10.93	10.79
PSAO	Q949Q5	Photosystem I subunit O	10.42	10.61
PSBT	P31336	Photosystem II 5 kDa protein, chloroplastic	10.35	10.40
PSBR	P06183	Photosystem II 10 kDa polypeptide, chloroplastic	11.50	11.23
PSBY	P80470	Photosystem II core complex proteins psbY, chloroplastic	10.39	9.83
psbA	Q7FNT3	Photosystem II protein D1	7.07	6.06
PSY	P49293	Phytoene synthase, chloroplastic	1.85	3.84
CURT1A	O04616	Protein CURVATURE THYLAKOID 1A, chloroplastic	9.37	9.38
SPA3	Q9LJR3	Protein SPA1-RELATED 3	2.61	4.04

Table S14. Twenty-nine genes involved in polynucleotide metabolism at VGS and EFS vs. GS.

Gene name	SwissProt ID	Protein name	log_2FC (VGS vs. GS)	log_2FC (EFS vs. GS)
**DNA replication**				
NRPB11	Q38859	DNA-directed RNA polymerases II, IV and V subunit 11	2.24	− 10.83
LIG1	Q42572	DNA ligase 1	− 10.99	− 10.99
ENDO4	F4JJL0	Endonuclease 4	− 1.23	− 1.40
gag-pol	P08361	Gag-Pol polyprotein	− 1.07	− 2.14
gag-pro-pol	P07572	Gag-Pro-Pol polyprotein	− 1.46	− 1.46
ORC5	Q6EWX0	Origin of replication complex subunit 5	1.18	1.74
ORC6	Q9ZVH3	Origin of replication complex subunit 6	1.24	2.45
RTEL1	A0A0P0V4R0	Regulator of telomere elongation helicase 1 homolog	1.07	1.15
top6A	O05208	Type 2 DNA topoisomerase 6 subunit A	− 1.02	− 1.29
**DNA integration**				
pol	Q8I7P9	Retrovirus-related Pol polyprotein from transposon opus	− 1.36	− 1.45
RE1	Q94HW2	Retrovirus-related Pol polyprotein from transposon RE1	− 3.09	− 3.72
R2	Q03274	Retrovirus-related Pol polyprotein from type-1 retrotransposable element R2	1.76	1.39
**DNA recombination**				
Pol	P11369	LINE-1 retrotransposable element ORF2 protein	− 1.81	− 1.19
**DNA repair**				
BARD1	F4I443	BRCA1-associated RING domain protein 1	− 13.14	− 8.35
EXO1	Q8L6Z7	Exonuclease 1	1.47	2.89
**RNA processing**				
XRN3	Q9FQ03	5'-3' exoribonuclease 3	11.92	11.53
CSP41B	Q9SA52	Chloroplast stem-loop binding protein of 41 kDa b, chloroplastic	9.59	9.34
At2g20020	Q9SL79	CRS2-associated factor 1, chloroplastic	9.54	10.11
PCMP-H12	Q9LN01	Pentatricopeptide repeat-containing protein At1g08070, chloroplastic	− 1.10	− 1.30
PCMP-H6	P93011	Pentatricopeptide repeat-containing protein At2g33760	3.68	3.32
At4g34730	O65693	Probable ribosome-binding factor A, chloroplastic	− 10.94	− 3.99
PEL1	Q9ZT87	Protein PELOTA 1	9.38	8.11
TE1	O65001	Protein terminal ear1	− 1.61	3.17

Os05g0571100	Q65XL5	Protein WHAT'S THIS FACTOR 1 homolog, chloroplastic	− 1.17	− 1.07
**RNA replication**				
RDRP	O15925	RNA-directed RNA polymerase	− 1.23	− 1.81
**Others**				
CID11	Q9LPI5	Polyadenylate-binding protein-interacting protein 11	1.17	1.85
PHN1	Q69VD5	Protein argonaute PNH1	− 1.04	1.26
DOGL3	Q58FV0	Protein DOG1-like 3	− 1.43	− 3.34
FRS5	Q9SZL8	Protein FAR1-RELATED SEQUENCE 5	− 2.38	− 2.42

Table S15. Twenty-three genes involved in protein metabolism at VGS and EFS vs. GS.

Gene name	SwissProt ID	Protein name	log_2FC (VGS vs. GS)	log_2FC (EFS vs. GS)
**Ribosomal protein**				
RPS16C	Q42340	40S ribosomal protein S16-3	9.49	9.98
RPS17	P49215	40S ribosomal protein S17	− 8.31	− 8.31
RPL22B	Q9M9W1	60S ribosomal protein L22-2	10.90	10.87
RPL9B	P49209	60S ribosomal protein L9-1	5.27	5.71
rnhA	F9VN79	Ribonuclease HI	− 1.88	− 2.02
RPL3	O96774	Ribosomal protein L3	7.23	8.45
**Protein ubiquitination**				
At2g24240	Q9ZUH1	BTB/POZ domain-containing protein At2g24240	2.77	3.22
At5g03250	Q9LYW0	BTB/POZ domain-containing protein At5g03250	1.08	1.17
At4g11680	Q93Z92	E3 ubiquitin-protein ligase At4g11680	11.39	10.26
ATL43	Q5EAE9	RING-H2 finger protein ATL43	2.52	1.69
ATL79	Q9FGJ6	RING-H2 finger protein ATL79	− 1.03	− 1.38
**Protein folding**				
CALR	O81919	Calreticulin	1.24	1.30
PBP1	O04314	PYK10-binding protein 1	5.06	5.25
**Protein biosynthetic process**				
fusA2	I1K0K6	Elongation factor G-2, chloroplastic	8.95	8.96
TUFB1	P46280	Elongation factor Tu, chloroplastic	9.10	8.74
IF5A	Q9AXJ4	Eukaryotic translation initiation factor 5A	− 4.36	− 5.48
**Protein catabolic process**				
MMP2	Q9GLE5	72 kDa type IV collagenase	− 3.85	− 5.07
VIT_05s0020g01830	A7NW79	CASP-like protein 1D1	− 1.67	− 4.50
COL1A2	P02465	Collagen alpha-2(I) chain	− 4.02	− 6.03
ASY1	F4HRV8	Meiosis-specific protein ASY1	1.01	3.55
PNG1	Q7F0R1	Peptide-N(4)-(N-acetyl-beta-glucosaminyl)asparagine amidase	− 2.60	− 2.75
UBP12	Q9FPT1	Ubiquitin C-terminal hydrolase 12	4.16	3.75
UREG	O64700	Urease accessory protein G	1.29	6.65

Table S16. Thirty-one genes involved in stress response at VGS and EFS vs. GS.

Gene name	SwissProt ID	Protein name	log_2FC (VGS vs. GS)	log_2FC (EFS vs. GS)
**Hydrogen peroxide catabolic process**				
CAT2	P30567	Catalase isozyme 2	10.60	10.13
PER12	Q96520	Peroxidase 12	1.13	1.12
PER19	O22959	Peroxidase 19	− 1.46	− 3.67
PER42	Q9SB81	Peroxidase 42	− 9.62	− 9.62
PER69	Q96511	Peroxidase 69	9.79	10.15
RBOHE	O81211	Respiratory burst oxidase homolog protein E	1.41	1.66
**Response to heat and cold**				
HSP17.7	P19242	17.1 kDa class II heat shock protein	3.76	5.41
HSP70-1	P22953	Heat shock 70 kDa protein 1	8.51	9.57
ERD14	P42763	Dehydrin ERD14	8.87	9.65
ERD15	Q39096	Protein EARLY RESPONSIVE TO DEHYDRATION 15	8.96	9.01
**Others**				
CDR1	Q6XBF8	Aspartic proteinase CDR1	− 1.83	− 1.59
ML3	P82683	Beta-galactoside-specific lectin 3	− 3.03	− 9.00
BIG5	F4IXW2	Brefeldin A-inhibited guanine nucleotide-exchange protein 5	− 9.42	− 1.90

KRP1	O81831	Calcium-binding protein KRP1	9.37	9.91
CFB	P81187	Complement factor B	− 9.76	− 9.76
DEF	A3FPF2	Defensin-like protein	3.24	8.68
PI206	P13240	Disease resistance response protein 206	1.13	2.26
ephB	I6YC03	Epoxide hydrolase B	2.05	1.45
At5g07800	Q9FF12	Flavin-containing monooxygenase FMO GS-OX-like 9	2.10	3.73
HIPP37	A2RVM8	Heavy metal-associated isoprenylated plant protein 37	1.58	3.66
IQM3	Q9LFA4	IQ domain-containing protein IQM3	− 1.28	− 3.27
JOX1	Q9SRM3	Jasmonate-induced oxygenase 1	− 1.03	− 5.49
MO3	Q9FLC2	Monooxygenase 3	− 1.67	− 3.08
ATL24	Q8LBA0	NEP1-interacting protein-like 2	7.23	10.95
N/A	Q41350	Osmotin-like protein	1.37	3.51
PTX3	Q0VCG9	Pentraxin-related protein PTX3	− 10.80	− 10.80
EXO	Q9ZPE7	Protein EXORDIUM	9.02	9.31
EXL2	Q9FE06	Protein EXORDIUM-like 2	− 1.29	− 1.40
DLO2	Q9ZSA7	Protein DMR6-LIKE OXYGENASE 2	− 2.25	− 4.25
TSPO	O82245	Translocator protein homolog	− 1.31	− 2.69
AMP2-2	Q9SPL4	Vicilin-like antimicrobial peptides 2-2	− 3.33	− 4.55

Table S17. Sequences of primer used in qRT-PCR validation.

Genes	Sequences (5' to 3')	Amplicon size (bp)
ACT	Forward: CTAAACCGCTTGTTGCTGGC<br>Reverse: GGGGAGCTCACACGAAAGAT	104
**Polysaccharide biosynthesis (12)**		
BGLU23	Forward: TCGTCGGCAAGCGGTATTTC<br>Reverse: CGACAACCTAACGACGATGC	106
GAPA	Forward: GATTGGACGGATAGCCCACG<br>Reverse: TGTCATCCGGCGCAATTCT	134
INVA	Forward: TGGTGATGGTTAGACTGGTTG<br>Reverse: TTAACCGTTTCCACACTGCC	105
FBA2	Forward: TGTTGAGGTGGATGTGTGGT<br>Reverse: GCCTTGCCGTAATAGTCTCAC	183
At3g55800	Forward: AATGGTGGGCAATAAGCAGG<br>Reverse: TTCGGCCTATATGCACCCTC	114
BXL5	Forward: AACCCTCAGCGGCTAATAAGG<br>Reverse: ACAACGCCTGGACTTCATCTT	159
TBL31	Forward: GAGGGAGGGTTAGGCATTCAC<br>Reverse: GTTTTGGTGTCCCAACTGCTC	149
XTH9	Forward: ATTGCCCAGCACTCACTACC<br>Reverse: GGTACGCCTGAGAATAGGGC	114
TPPF	Forward: ACTTCTCAAGTTGCCCCACC<br>Reverse: GACTCGAAGAGTGGGAGTGC	187
PAE8	Forward: ACAAGTGTGCTCGGGAAAGG<br>Reverse: AGTTGACCTCGTCGTGTGTT	111
PME7	Forward: TCTGTGCTGGAAAGGCAAATC<br>Reverse: ATCACCATCACCACATGCAC	142
PMEI10	Forward: GGCAGCCAGATTGACTCTTTTC<br>Reverse: GTTGGGTAATGGGTGGCACG	100
**Flavonoid biosynthesis (11)**		
4CLL1	Forward primer: TGTATTGAGGTGGATGTGTGGT<br>Reverse primer: ATCGAACAACCTCAAATGGTTCT	124
4CLL6	Forward: CTCAAAGGACAAGGAGGCGT<br>Reverse: TGATCCACCACTCATCTCCG	195
HST	Forward: CCAGAAAATGGGCCTGACAC<br>Reverse: CACAGTCATCTGCGAGGTTT	139
CHI3	Forward: GGTCATCCAGTAACGAACGC<br>Reverse: ATTCAGCGTCTGCTAACCGC	104
CAD9	Forward: TCCACTCGTCTAGGCACTCT<br>Reverse: TGTCTCCGGCCAGCTTTATG	169
CYP714C2	Forward: TGACCATGCTAAAAGAGTGGTG<br>Reverse: AATTGAGTTACGGCGGGGC	111
CYP93B1	Forward: CGCTCTGAAGGCGAGTAATG<br>Reverse: ATCGGAATCGAATCGTGTGTT	178

F6′H1	Forward: ATCCACCAAAATGGCACTCC<br>Reverse: ATTGACGAGACTAGAAGTGGCG	121
UGT84A13	Forward: CCTCGACTGTTACATGCCCG<br>Reverse: GAGAGGAGGTGTTCGTCGTC	161
UGT87A1	Forward: TGACCTAGATTGCCACCCCT<br>Reverse: AAGCACCCCGAAATACCGTT	200
UGT94E5	Forward: TGGTTTGCCCATAGCACCTT<br>Reverse: CGGCACTACACATTTGACCT	161
**Cell growth and flower development (8)**		
AC58	Forward: ATTTGCCCCATGCTTCCACT<br>Reverse: TGTGCAAGTGGTTGAAGATCC	122
CYCP3-1	Forward: TCCTCGACCCTACTTCTGCT<br>Reverse: AGTGGTGTCGCAAATGTGGT	167
MIZ1	Forward: TGCCACAATGGCCCAAGATT<br>Reverse: TAGTGGTCCACCCTTACCGC	200
TBB7	Forward: TTCCGAGTCCTGCTTGTGTT<br>Reverse: GCCCCATATGTGATATGAGTAAAGC	131
AP1	Forward: ACCATCGAGACCGACACATC<br>Reverse: TCCAAGTCCGAATAGCCTCG	160
AMP1	Forward: CATCGTCACCGCTAGTCTCC<br>Reverse: CCGGGATTGGCATAGTTGGT	165
TKPR2	Forward: CAAGGGTTCAAATGAGGGTGG<br>Reverse: AATTTTCTCCAGCACTCCGT	162
HAT	Forward: ATGTGGGCAGAGGACTGATT<br>Reverse: GCTGAGAATCGCCCATATTGTG	146
**Hormone Biosynthesis and Signaling (15)**		
YUC8	Forward: TGATCTCCTAGGTGCGCTTC<br>Reverse: ATTATACGCCGTCGCCATCC	114
AIPT	Forward: GAGGTTCGCTTTTGCTGGAC<br>Reverse: ACACTGATCCGCCTGTTACC	179
LOG5	Forward: GACCGCCCTGAGGGTAATTC<br>Reverse: GAGGAGGAGCGTACAACCAC	186
LE	Forward: CTACGGCGTCACGAATTGC<br>Reverse: CCACGACGCGAAAAATTGTG	117
ACO1	Forward: CCCGTGCAAATACCACAAGC<br>Reverse: TTGGATTTGGTCGGAGGAGC	172
ACS1	Forward: ACAGCAAGGAGCACGGATAG<br>Reverse: AAACACGGTTGTGTGACTCG	121
CYP707A6	Forward: GTCGGTTTTTCTGGTTCGGTT<br>Reverse: ACCACGACTCGACCAACCT	102
AUX22D	Forward: TTTTGGTTGCTTGGCTCACG<br>Reverse: TAAGCTTTGGACTTGCCGCT	108
SAUR71	Forward: GATAGTCCCGGAGGCAAGGT<br>Reverse: ACCTATGCACGAAAGTCGAGG	100
GH3.6	Forward: AGTCGACCCTTTTGGAGGAC<br>Reverse: GCTCGTTCCACATTCCCTCT	170
AHK4	Forward: CAAGAACCCAAGGGCCAAGT<br>Reverse: CGGAAAAGCACCCAGTCTCC	100
JOX2	Forward: CCTTCAGTCCAAAAGTGTGGC<br>Reverse: GTCGAGTCAACCAAGGGATGT	149
AIL1	Forward: ATGGATGTCGGTGAGAGAGGT<br>Reverse: CTATCTGTCACTGGGGCAACT	148
ERF010	Forward: AGGGTTTGATTTTCCAGGTTGG<br>Reverse: TGTTCCGCCACTGTTCTACC	139
ERF114	Forward: AGGAGGTCCACGAAGGAGTT<br>Reverse: ATCGGCGAACCTTTGTCACT	150
**Transcription factor (12)**		
**Flavonoid biosynthesis (6)**		
MYB2	Forward: TAAGGAATAAGAACAACGGGGC<br>Reverse: AGCGATGATGCACGAATGTT	100
MYB14	Forward: TGAGGATAGTGCTAGAGGCAAC<br>Reverse: AGGGGTAAACTATGCCGATGG	100
MYB83	Forward: TGCGTTGTTGGGCTCTTTTAC<br>Reverse: GTGGTGGTTGTAATGGGTGG	192

BHLH52	Forward: TTGGCATGAGATGAGCTGCG<br>Reverse: GGTCCCAGACAGCCAGAATG	101
WRKY6	Forward: TTCAACTGGCCTCCTCGAAC<br>Reverse: CAACCTTTTCCACCGATGCC	190
WRKY53	Forward: TTGGGCGAATTTGTCCAGTC<br>Reverse: GGGGGCTTAGACTAGGGTTG	125
WRKY72	Forward: GGCTCGGTATTATCCTCCGC<br>Reverse: CGACAGCAACTCCGCAAAAT	132
**Growth and development (6)**		
DREB2D	Forward: GGTGTTCGTCGTGTACGTCT<br>Reverse: TCGTCGTTCCCAATCTCGTC	195
At2g01810	Forward: TCGGCACCCCTAAAAACTGC<br>Reverse: AGGGTGAGGAGTGCAAAGAC	109
TCP9	Forward: AGCTTCTTTGGATTGCGCCT<br>Reverse: TGGCTCTGACACCATGAAGAC	130
UPB1	Forward: GAAGGGGGTTCGGTTGGTC<br>Reverse: CGAGCAGTAGCCCAAACAAT	195
AHL20	Forward: TCACCAGGCGTGAATTGTCT<br>Reverse: GAGAAGGACAAGAGGTGCGT	200
**Polysaccharide transport (6)**		
SWEET5	Forward: CTCCTTCGCCCAGCCATTAC<br>Reverse: AGCAACATGAGGCCAAAAGTC	192
slc37a2	Forward: GGTGGCATCGTAGCTGGATT<br>Reverse: TCCATTCACGAGAGCACCAC	171
At5g55950	Forward: TGAGTTGGTACCCTCTTGGTG<br>Reverse: TCAGGACCTCCTCCTCGATAC	104
ABCB2	Forward: AAAGGTTCCACATGCCTCCAC<br>Reverse: ACTACTTCCCAGCGTTCCTT	132
ABCG1	Forward: TTCTGACGGACTTCTAAAACTCTT<br>Reverse: GTATGCGTGCTGGCCTTTTC	156
ABCG22	Forward: GGCGCTCGTCATAGATGGTT<br>Reverse: AACTTCCTTCCAGTGTCCCG	161
